# Dataset of traffic accidents in motorcyclists in Bogotá, Colombia

**DOI:** 10.1016/j.dib.2022.108461

**Published:** 2022-07-16

**Authors:** Holman Ospina-Mateus, Shyrle Berrio Garcia, Leonardo Quintana Jiménez, Katherinne Salas-Navarro

**Affiliations:** a Department of Industrial Engineering, Universidad Tecnológica de Bolívar, Cartagena, Colombia; b Department of Industrial Engineering, Pontificia Universidad Javeriana, Bogotá, Colombia; c Department of Productivity and Innovation, Universidad de la Costa, Barranquilla, Colombia

**Keywords:** Motorcyclists, Traffic accidents, Road injuries, Road fatalities, Crashes, Collision

## Abstract

According to the World Health Organization, in 2016, Colombia obtained the tenth position worldwide, the third in the continent and the second in South America, according to the accident rate of 9.7 motorcycle fatalities per 100,000 populations. Between 2012 and 2021, the number of deceased and injured motorcyclists among all road users was 50%, with an annual average of 3140 fatal victims and 20,800 injured victims. Bogotá, Cali, and Medellín were the cities with the most accidents. In Bogota in 2017, the deaths of motorcyclists on the roads were around 32% of the road actors. This data article presents the dataset used to analyze and predict the severity of motorcyclist road accidents in Bogota in the article entitled “Extraction of decision rules using genetic algorithms and simulated annealing for prediction of severity of traffic accidents by motorcyclists” [1]. The data set was consolidated from the registration of 175,245 traffic accidents and the report of 337,828 road actors involved in crashes in Bogotá between January 2013 and February 2018. The data was compiled, processed, and enriched with additional information about infrastructure and weather conditions. The data corresponds to 35,693 motorcyclist traffic accidents, represented by 28 variables, and classified into five categories: road actors, motorcyclists and individuals involved, weather conditions and timing, road conditions and location and characteristics of the accident. The data on motorcyclist traffic accidents opens up a scenario to deepen and compare road safety in Latin America, where studies on vulnerable road users are limited. According to severity, the data on motorcycle traffic accidents recorded 28% with material damage, 69% with injured and 3% with fatal victims.

## Specifications Table

**Table:** 

Subject	Safety Research, Transportation Engineering.
Specific subject area	Traffic accidents, road safety, traffic crash, vulnerable road users.
Type of data	Table.
How the data were acquired	The data correspond to motorcycle traffic accidents recorded by traffic agents in Bogotá. The records contain information on the location and time, people, vehicles involved and a preliminary description of the event. Additionally, the data was correlated with the registry of victims in the health and care system, where sociodemographic data and the patient's health triage were obtained. All data was provided by the Secretariat of Mobility and Transit of Bogotá, a government entity for road safety. The data was obtained with labels and anonymized, respecting Colombia's legal and ethical provisions. In addition, the data set was enriched with the historical conditions of the infrastructure (roads/pavement) developed by the Governmental Institution of Urbanism of Bogota. The data related to the climatic conditions of Bogota were also added from the historical records of the Institute of Hydrology, Meteorology and Environmental Studies of Colombia for Bogotá. The organization and classification of the data were developed in MS Excel and the statistical software IBM SPSS (version 25.0).
Data format	Raw and analyzed.
Description of data collection	The data set was consolidated from the registration of 175,245 traffic accidents and the report of 337,828 road actors involved in crashes in Bogotá between January 2013 and February 2018. The data corresponds to 35,693 motorcyclist traffic accidents, represented by 28 variables, and classified into five categories: (1) road actors involved in the event (car, bus, bicycle, motorcycle, and pedestrian); (2) motorcyclists and individuals involved (number, age, and gender); (3) weather conditions and timing (day, month, hours, holidays, light conditions, and precipitation); (4) road conditions and location (place, address and status of the road network); (5) characteristics of the accident (number of people injured, uninjured, dead, and type of accident).
Data source location	Bogotá D.C, Colombia Reports of road events in Bogotá and related victims were provided (digital files) by the Secretariat of Mobility and Transit of Bogotá. The road conditions were obtained from the Governmental Institution of Urbanism of Bogota [2], and weather conditions were obtained from the Institute of Hydrology, Meteorology and Environmental Studies of Colombia [3].
Data accessibility	Repository name: Mendeley Data Data identification number: 10.17632/rm9m7ycp3r.1 Direct URL to data: http://dx.doi.org/10.17632/rm9m7ycp3r.1
Related research article	Ospina-Mateus, H., Quintana Jiménez, L. A., Lopez-Valdes, F. J., Berrio Garcia, S., Barrero, L. H., & Sana, S. S. (2021). Extraction of decision rules using genetic algorithms and simulated annealing for prediction of severity of traffic accidents by motorcyclists. Journal of Ambient Intelligence and Humanized Computing, 12(11), 10051-10072. doi: 10.1007/s12652-020-02759-5

## Value of the Data

• The data can be used to predict the conditions and factors associated with a motorcyclist traffic accident in Bogotá (Colombia) according to severity (material damage, injuries, and deaths).
• The availability of data related to traffic accidents in motorcyclists is limited in Latin America.
• The data can be analyzed comparatively with traffic crashes from other locations to contrast the behavior of motorcyclists on the road.
• The data can help identify the causality of the motorcyclist traffic accident and thus define countermeasures to prevent injuries and fatalities on the roads.
• Motorcyclist traffic accident data includes information on pavement/road conditions and weather conditions related to the time and location of the crash/collision.
• The data set consolidates a relevant source for developing motorcyclists' road safety studies.

## 1 Data Description

The data presented in this brief article predicted the severity of traffic accidents in motorcyclists in Bogota, developed in the study by Ospina-Mateus, et al. [1]. In this study, data mining and machine learning techniques were applied to extract decision rules that predict motorcyclists' severity of traffic accidents. The data contains traffic accidents involving motorcyclists between January 2013 and February 2018 in Bogota, Colombia. The data set was extracted from 175,245 traffic accidents and 337,828 reports of road actors involved in crashes. In total, 35,693 motorcyclist accidents were consolidated.

The dataset was classified according to the accident's severity: material damage, injuries, and fatalities. In total, 28 variables were defined for each of the events. These variables were classified into five categories; road actors, motorcyclists and individuals involved, weather conditions and timing, location and road conditions, and accident characteristics. The data files (reads in Excel format) were presented in Tables 1 and 2, respectively, deposited in Mendeley Data. All variables present in each event were categorically defined in the dataset. The categorization of each variable is indicated and explained in Table 1. Table 2 contains the compilation of all the information.

## 2 Experimental Design, Materials and Methods

The dataset considered the variables in 5 groups. The “road actors” variables indicate the users (car/bus, bicycle, motorcycle, pedestrian) involved in the accident and the crash interaction. The variables related to “motorcyclists and individuals” indicate the number of people involved, gender and age. The variables of weather conditions and timing variables considered specific conditions such as day/date, lighting (daylight/nightlight), type of day (weekdays/weekends), month (trimester) and climatic aspects. The climatic conditions were consulted with the Institute of Hydrology, Meteorology and Environmental Studies of Colombia (IDEAM [3]) for the date of the event with the level of rainfall (mm). The variables related to the condition of the road and the location correspond to cardinally locating each accident and indicating the type of road and its quality. The information on the quality of the road network was provided by the Institute of Urbanism of Bogota ((IDU-UAERMV) [2]). Finally, in the last group of variables, the characteristics of the accident were indicated. These characteristics include the type of accident and the number of victims involved, whether uninjured, wounded, or dead. Table 3 contains the dataset with the variables and the severity of the road event. According to the data and severity, 28% correspond to events with material damage, 69% with injuries, and 3% with accidents with fatalities.

**Table 3:** Summary statistics of the dataset.

				Only damages		Accident with injuries		Accident with fatalities	
Variables	Description	Total	%	Total	%	Total	%	Total	%
**Number of Cars and buses**	Without Vehicle	11626	32.6	355	3.6	10760	43.5	511	47.8
	One Vehicle	22244	62.3	8593	86.7	13176	53.3	475	44.4
	Two Vehicles	1618	4.5	860	8.7	676	2.7	82	7.7
	More than two vehicles	205	0.6	104	1.0	99	0.4	2	0.2

**Number of Motorcyclists**	One motorcycle	33751	94.6	9538	96.2	23186	93.8	1027	96.0
	Two motorcycles	1864	5.2	355	3.6	1470	5.9	39	3.6
	More than two motorcycles	78	0.2	19	0.2	55	0.2	4	0.4

**Number of Cyclists**	Without bicycles	34177	95.8	9843	99.3	23294	94.3	1040	97.2
	One bicycle	1511	4.2	68	0.7	1414	5.7	29	2.7
	More than two bicycles	5	0.0	1	0.0	3	0.0	1	0.1

**Number of Pedestrians**	Without pedestrian	28065	78.6	9910	100.0	17418	70.5	737	68.9
	One pedestrian	7136	20.0	1	0.0	6820	27.6	315	29.4
	More than two pedestrians	492	1.4	1	0.0	473	1.9	18	1.7

**Number of Road Actors**	One road actor	3067	8.6	301	3.0	2601	10.5	165	15.4
	Two road actors	32043	89.8	9594	96.8	21561	87.3	888	83.0
	Three road actors	581	1.6	17	0.2	547	2.2	17	1.6
	Four road actors	2	0.0	0	0.0	2	0.0	0	0.0

**Relationship of Road Actors**	Solo Motorcycle	3067	8.6	301	3.0	2601	10.5	165	15.4
	Motorcycle-Vehicle	23546	66.0	9540	96.2	13464	54.5	542	50.7
	Motorcycle-Pedestrian	7123	20.0	0	0.0	6802	27.5	321	30.0
	Motorcycle-Bicycle	1374	3.8	54	0.5	1295	5.2	25	2.3
	Motorcycle-Vehicle-Pedestrian	441	1.2	2	0.0	427	1.7	12	1.1
	Motorcycle-Vehicle-Bicycle	78	0.2	15	0.2	58	0.2	5	0.5
	Motorcycle-Bicycle-Pedestrian	62	0.2	0	0.0	62	0.3	0	0.0
	All actors	2	0.0	0	0.0	2	0.0	0	0.0
**Gender of Involved**	Masculine	32858	92.1	9240	93.2	22623	91.6	995	93.0
	Female	2290	6.4	510	5.1	1722	7.0	58	5.4
	Male/female	239	0.7	46	0.5	190	0.8	3	0.3
	Not defined	306	0.9	116	1.2	176	0.7	14	1.3

**Number of Men**	Without Man	628	1.8	112	1.1	491	2.0	25	2.3
	One man	8598	24.1	1686	17.0	6622	26.8	290	27.1
	Two men	23011	64.5	7265	73.3	15152	61.3	594	55.5
	Three men	3069	8.6	743	7.5	2193	8.9	133	12.4
	Four men or more	387	1.1	106	1.1	253	1.0	28	2.6

**Number of Women**	Without Woman	24427	68.4	8186	82.6	15475	62.6	766	71.6
	One woman	9960	27.9	1617	16.3	8072	32.7	271	25.3
	Two women	1201	3.4	102	1.0	1071	4.3	28	2.6
	Three women or more	105	0.3	7	0.1	93	0.4	5	0.5

**Motorcyclists Age (years)**	(0-19)	2146	6.0	511	5.2	1563	6.3	72	6.7
	(20-39)	28914	81.0	8000	80.7	20065	81.2	849	79.3
	(40-59)	4228	11.8	1271	12.8	2816	11.4	141	13.2
	(60-79)	205	0.6	55	0.6	145	0.6	5	0.5
	(79>)	200	0.6	75	0.8	122	0.5	3	0.3

**Driver-other Age (years)**	(0-19)	2237	6.3	161	1.6	2052	8.3	24	2.2
	(20-39)	16140	45.2	4981	50.3	10745	43.5	414	38.7
	(40-59)	11291	31.6	3775	38.1	7227	29.2	289	27.0
	(60-79)	3087	8.6	581	5.9	2353	9.5	153	14.3
	(79>)	2938	8.2	414	4.2	2334	9.4	190	17.8

**Light condition**	Daytime	23729	66.5	7234	73.0	15930	64.5	565	52.8
	Night	11964	33.5	2678	27.0	8781	35.5	505	47.2

**Time (hours)**	(00:00 - 06:00)	2610	7.3	496	5.0	1928	7.8	186	17.4
	(06:00-12:00)	12321	34.5	3694	37.3	8327	33.7	300	28.0
	(12:00- 18:00)	11408	32.0	3540	35.7	7603	30.8	265	24.8
	(18:00- 24:00)	9354	26.2	2182	22.0	6853	27.7	319	29.8
**Day**	Monday	4843	13.6	1297	13.1	3412	13.8	134	12.5
	Tuesday	5538	15.5	1678	16.9	3706	15.0	154	14.4
	Wednesday	5553	15.6	1608	16.2	3772	15.3	173	16.2
	Thursday	5350	15.0	1469	14.8	3737	15.1	144	13.5
	Friday	5715	16.0	1615	16.3	3940	15.9	160	15.0
	Saturday	5259	14.7	1409	14.2	3674	14.9	176	16.4
	Sunday	3435	9.6	836	8.4	2470	10.0	129	12.1

**Type of Day**	Holiday	1110	3.1	257	2.6	814	3.3	39	3.6
	Working day	34583	96.9	9655	97.4	23897	96.7	1031	96.4

**Month**	January–March	6263	17.5	1728	17.4	4364	17.7	171	16.0
	April–June	9015	25.3	2491	25.1	6275	25.4	249	23.3
	July–September	8690	24.3	2386	24.1	6058	24.5	246	23.0
	October–December	11725	32.8	3307	33.4	8014	32.4	404	37.8

**Day of the Week**	During the week	26999	75.6	7667	77.4	18567	75.1	765	71.5
	Weekend	8694	24.4	2245	22.6	6144	24.9	305	28.5

**Weather Conditions (Prec. mm)**	(0-2)	1056	3.0	353	3.6	664	2.7	39	3.6
	(2-15)	3898	10.9	1071	10.8	2687	10.9	140	13.1
	(15-30)	1152	3.2	341	3.4	775	3.1	36	3.4
	(30-60)	12569	35.2	3429	34.6	8761	35.5	379	35.4
	(<60)	17018	47.7	4718	47.6	11824	47.8	476	44.5

**Road Type**	Main Road	25675	71.9	7632	77.0	17227	69.7	816	76.3
	Secondary Road	10018	28.1	2280	23.0	7484	30.3	254	23.7

**Location**	North	9468	26.5	2519	25.4	6684	27.0	265	24.8
	Middle East	9677	27.1	2642	26.7	6779	27.4	256	23.9
	Southeast	10011	28.0	2609	26.3	7045	28.5	357	33.4
	South	6537	18.3	2142	21.6	4203	17.0	192	17.9
**State of Main Road**	Regular	2421	6.8	410	4.1	1912	7.7	99	9.3
	Acceptable	24732	69.3	6412	64.7	17538	71.0	782	73.1
	Good	6052	17.0	2103	21.2	3814	15.4	135	12.6
	Excellent	2488	7.0	987	10.0	1447	5.9	54	5.0

**State of Intermediate Road**	Regular	12635	35.4	4147	41.8	8168	33.1	320	29.9
	Acceptable	23058	64.6	5765	58.2	16543	66.9	750	70.1

**State of Local Road**	Regular	34725	97.3	9710	98.0	23975	97.0	1040	97.2
	Acceptable	968	2.7	202	2.0	736	3.0	30	2.8

**Accident Class**	Collision	27346	76.6	9846	99.3	16865	68.2	635	59.3
	Falling from the vehicle	52	0.1	0	0.0	51	0.2	1	0.1
	Run Over	7124	20.0	1	0.0	6805	27.5	318	29.7
	Dump	870	2.4	49	0.5	709	2.9	112	10.5
	Fire	1	0.0	0	0.0	1	0.0	0	0.0
	Self-injury	182	0.5	0	0.0	180	0.7	2	0.2
	Others	118	0.3	16	0.2	100	0.4	2	0.2

**Involved Injured**	No uninjured	5057	14.2	2	0.0	4717	19.1	338	31.6
	One uninjured	18886	52.9	204	2.1	18075	73.1	607	56.7
	Two uninjured	10446	29.3	8582	86.6	1750	7.1	114	10.7
	Three uninjured or more	1304	3.7	1124	11.3	169	0.7	11	1.0

**Injured Victim**	No injured	10710	30.0	9903	99.9	100	0.4	707	66.1
	One injured	17316	48.5	7	0.1	17008	68.8	301	28.1
	Two injured	6602	18.5	1	0.0	6546	26.5	55	5.1
	Three injured	1065	3.0	1	0.0	1057	4.3	7	0.7

**Dead Victim**	Without dead	34489	96.6	9912	100.0	24575	99.4	2	0.2
	One dead	1175	3.3	0	0.0	134	0.5	1041	97.3
	Two dead or more	29	0.1	0	0.0	2	0.0	27	2.5

**Injured or Dead Victim**	No Recognized	35408	99.2	9818	99.1	24537	99.3	1053	98.4
	One injured/dead	283	0.8	93	0.9	173	0.7	17	1.6
	Two injured/dead or more	2	0.0	1	0.0	1	0.0	0	0.0

## Ethics Statements

The data and information related to motorcycle traffic accidents were received formally anonymized, guaranteeing the rights to privacy of humans involved in road events. The primary information was provided by the Secretariat of Mobility and Transit of Bogotá, a Colombian government entity committed to guaranteeing ethical and legal provisions in the use of information.

## CRediT authorship contribution statement

**Holman Ospina-Mateus:** Conceptualization, Methodology, Data curation, Software, Writing – review & editing. **Shyrle Berrio Garcia:** Data curation. **Leonardo Quintana Jiménez:** Supervision. **Katherinne Salas-Navarro:** Writing – review & editing, Validation.

## Declaration of competing Interest

The authors declare that they have no known competing financial interests or personal relationships that could have appeared to influence the work reported in this paper.

## Data Availability

Dataset of road crashes in motorcyclists in Bogotá (Original data) (MENDELEY).

## References

[1] Ospina-Mateus H., Quintana Jiménez L.A., Lopez-Valdes F.J., Berrio Garcia S., Barrero L.H., Sana S.S. (2021). Extraction of decision rules using genetic algorithms and simulated annealing for prediction of severity of traffic accidents by motorcyclists. J. Ambient Intell. Humaniz. Comput..

[2] (IDU-UAERMV) (2020). in: Road Prioritization Model Road Maintenance Unit.

[3] IDEAM (2017). in: Instituto de Hidrología, Meteorología y Estudios Ambientales.

